# Power, Reliability, and Heterogeneous Results

**DOI:** 10.1371/journal.pmed.0020386

**Published:** 2005-11-29

**Authors:** Ian Shrier

**Affiliations:** **1**McGill UniversityMontreal, QuebecCanada

## Abstract

N/A

I want to congratulate John P. A. Ioannidis on his thought-provoking Essay [1]. I have two comments.

In Corollary 1, he suggests that small sample sizes mean smaller power, and implies that larger studies with thousands of subjects are more likely to be true. I think it is important to stress that if the effect size is large (e.g., very small variance that is seen in physiological studies), then adequate power is obtained with small numbers. Furthermore, some would argue that exposing subjects to research risks unnecessarily (e.g., when fewer subjects would yield sufficient power) is unethical. Since the analysis is based on power, we should remember that larger is not always better.

In Corollary 4, Ioannidis argues that greater flexibility in designs, definitions, etc. means the results are less likely to be true. I agree that replication of all aspects of the study is more likely to yield consistent results, but this does not necessarily mean true results. Since we don't know a priori which methodological details are most appropriate (e.g., dose, timing, etc.), heterogeneous results from different designs is an important source of information and can lead to a new, more in-depth understanding of the subject—and sometimes even paradigm shifts. I agree with the accompanying Editorial [2] to the article that we need to distinguish between the validity of the data and the validity of the authors' conclusions.
